# Combining exercise and hypoxia for brain health: from elite athlete altitude training camps to clinical applications

**DOI:** 10.3934/Neuroscience.2026014

**Published:** 2026-06-01

**Authors:** Johannes Burtscher, Martin Burtscher, Katharina Hüfner

**Affiliations:** 1 Department of Psychiatry, Psychotherapy, Psychosomatics and Medical Psychology, University Hospital for Psychiatry II, Medical University of Innsbruck, Innsbruck, Austria; 2 Department of Sport Science, University of Innsbruck, Innsbruck, Austria

**Keywords:** physical activity, central nervous system, neurological diseases, psychiatric symptoms, hypoxia conditioning, intermittent hypoxia therapy

## Abstract

Regular physical exercise promotes overall and brain health and can improve cognition and mood. Similar effects on brain health have been observed after well-controlled intermittent exposures to low ambient oxygen (hypoxia), termed hypoxia conditioning, in several pilot studies. This raises the question of how exercising in hypoxia affects the brain. Among hypoxic exercise training forms are altitude training camps, which have been used systematically by athletes to improve physical performance for many decades, or exposure to simulated altitude/inspiratory hypoxia in laboratory settings, combined or not with exercise. Here, we explore the theoretical basis of overlaps and differences in brain health-related physiological responses to exercise and hypoxia. We present different types of altitude training undertaken by athletes, as well as hypoxia conditioning methods used to enhance physical performance and brain health and to reduce symptoms of neuropsychiatric diseases. We attempt to evaluate the potential of different altitude/hypoxia training types for brain health and indicate potential links to selected clinical applications of combined exercise and hypoxia. In conclusion, the effects of traditional types of altitude training on brain health and function have been poorly investigated, but various benefits of intermittent hypoxia conditioning have been demonstrated, indicating its potential for clinical applications in improving cognition and mental health. Remaining challenges include reducing the stigma surrounding the negative effects of severe hypoxia on the brain, standardizing terminology and approaches to therapeutic hypoxia, and selecting optimized protocols of therapeutic hypoxia exposures and combinations with exercise for specific general health-promoting or clinical applications.

## 1. Introduction

Regular exercise confers substantial health benefits for many different organs, including the brain [1],[2]. The mechanistic underpinnings of these health-promoting consequences of exercise are, however, not sufficiently understood, especially for organs and tissues that are not as directly impacted by physical activity as skeletal muscle and the cardiovascular system. However, regular exercise has the potential to sustain virtually all hallmarks of health [3], including immunity and inflammation regulation [4], mental health [5], and healthy brain aging [2].

We discuss the hypothesis that increased oxygen demand during exercise, particularly when performed in hypoxic conditions (reduced oxygen availability, such as occurring naturally at high altitude or mimicked in hypoxia chambers or by inspiring hypoxic gas mixtures), triggers beneficial adaptations throughout the body, including in the brain. Whether a stimulus with a harmful potential, like hypoxia, can be beneficial, in particular in an oxygen-dependent organ like the brain, has long been debated and is still being discussed [6]. Nowadays, a number of studies support positive outcomes of specific mild hypoxia interventions, sometimes referred to as “hypoxia conditioning”, on human health in general and to reduce symptoms of neuropsychiatric diseases in specific [7],[8]. Hypoxia conditioning commonly consists of highly controlled mild and often intermittent hypoxia exposures, combined with sufficient normoxic or hyperoxic regeneration phases. A crucial determinant of whether hypoxia is detrimental, for example, to the brain, or whether it induces adaptations that increase resilience and functional capacities, is the “hypoxic dose”, defined as the duration, intensity, frequency, and pattern of the hypoxia exposure; as long as the individual resilience is sufficient to manage the hypoxic stress, beneficial adaptations may occur. Another debated topic is whether the combination of exercise and ambient (inspiratory) hypoxia affords complementary or synergistic effects on brain health. Although a previous study failed to demonstrate the additional benefits of hypoxic exercise on cognitive function [9], a meta-analysis indicated synergistic effects [10]. It is important to note that different characteristics aside from the hypoxic dose (for example, age, the type of cognitive task, and exercise type and intensity) had a big effect on how exercise and hypoxia affected cognitive function in the available studies [10]. In addition, the timing of cognitive function testing plays a role. For example, in a study by Ando and colleagues [9], cognitive testing was conducted during hypoxia (18% or 15% oxygen). Unlike in the normoxic exercise group, no improvements in cognitive performance were observed. These results were recently replicated in a study, in which young adults had similar cognitive performance levels in 20.9%, 17.4%, 14.5%, and 12.7% oxygen conditions, although the perceived difficulty of the tests increased with hypoxia severity [11]. Conversely, studies have repeatedly demonstrated that testing following hypoxic conditioning leads to enhanced cognitive performance, as summarized in a recent systematic review [12]. The studies in that review included mainly older people (≥58 years), while the participants in the studies reporting no effects of hypoxia on cognition [9],[11] were young (18–30 years).

Long before hypoxia conditioning was systematically investigated in the context of brain health, hypoxia was employed by athletes for physical performance enhancement. As altitude training has long been an essential part of many athletes' training programs [13], it is of interest to take a closer look at the known underlying mechanisms and discuss how they may contribute to brain health. Moreover, it seems worthwhile to explore which additional protective mechanisms related to altitude training may contribute to general health, considering the multitude of clinical applications suggested for hypoxia conditioning regarding brain health, but also cardiometabolic health, for example, of overweight or obese adults, where combining exercise with inspiratory hypoxia has become a promising strategy [14]. Therefore, we review suggested mechanisms and the effectiveness of different altitude training variants, focusing on their effects on the brain, and compare them to strategies involving exercise and inspiratory hypoxia that have been applied in clinical/laboratory settings. We propose that, despite low levels of evidence for most such approaches individually (diverse hypoxia and exercise protocols, applications in different populations and clinical contexts), the large number of heterogeneous applications enables a preliminary comparison of different modalities and a relative assessment of advantages, safety, and risks of the variation of specific hypoxia parameters between the different approaches. Many recent and ongoing investigations have explored the therapeutic potential of hypoxia-based interventions on brain health. A comprehensive overview of the various applications and diverse protocols used for hypoxia conditioning is beyond the scope of this review. Therefore, we limit our analysis to selected applications related to findings from recent clinical trials, including cognitive function, Parkinson's disease, and stroke.

## 2. Methods

We searched PubMed and Google Scholar for articles linking altitude training and other hypoxic training and conditioning modalities with outcomes related to brain health and functions from inception to March 1, 2026. Supplementary searches were conducted through backward and forward citation chaining of relevant studies. Search keywords included altitude training, intermittent hypoxia, hypoxia conditioning, hypoxic training, hypoxia therapy, and brain health, cognition, neurological, and cerebral. We focused on clinical studies but also considered preclinical work and reviews. Regarding clinical applications of hypoxia, we primarily selected studies on recent or ongoing clinical trials investigating neurological and/or psychiatric diseases.

## 3. Overlaps of exercise and hypoxia responses

Before discussing the potential overlaps and synergies of hypoxic environments and exercising on the brain, and how those may be harnessed for clinical applications, it is worthwhile considering general physiological responses and adaptations to exercise and hypoxia in humans. Physiological and metabolic plasticity specifically following regular aerobic (endurance) exercise notably includes increased efficiency of systemic oxygen transport on one hand and changes in cellular oxygen delivery and utilization on the other hand [15]. Such responses are a logical result of reduced oxygen availability in tissues, which can be a consequence of elevated energetic and oxygen demand in tissues exposed to direct physical and metabolic exercise stress, mainly skeletal muscle and heart [16],[17]. To meet the high metabolic demands of intense exercise, oxygen is preferentially directed toward these tissues, leading to up to about 100-fold higher oxygen fluxes, for example, in working skeletal muscle [18]. In parallel, a comparably moderately increased cerebral blood flow of approximately 10%–30% [19] counteracts reductions of oxygen and substrate supply (including glucose) to the brain, one of the organs of the human body with the greatest oxygen consumption at (physical) rest. Thereby, the brain maintains its metabolic activity during exercise, despite the large re-allocation of blood and oxygen, especially to skeletal muscle. The regulation of cerebral blood flow by exercise is thought to constitute one important aspect of exercise that leads to positive effects on the brain [19]. Plastic adaptations of the cardiovascular system likely benefit virtually all organs.

Both hypoxia exposure [20] and exercise [21] lead to a ventilatory response counteracting reduced oxygen uptake or increased oxygen demand, respectively. Moreover, the hypoxic stress resulting from increased energetic demand and oxygen consumption in skeletal muscle also leads to the induction of hypoxia response pathways, for example, involving hypoxia-inducible factors (HIFs) [22],[23]. The organ-specific induction of HIFs, apart from blood and skeletal muscle, is not well understood in humans, especially in the brain. However, in animal models, the activation of HIFs during exercise has also been shown in remote tissues, including in the brain [24],[25]. It remains unknown how these hypoxia signals are induced in those tissues, but the redistribution of blood flow during exercise might play a role, as not only hypoxia per se but also changes in oxygen availability can lead to increased HIF levels and overall cellular responses reminiscent of hypoxia responses, as recently reviewed [26].

In addition, mechanical and metabolic stresses of exercise lead to the synthesis and release of signaling molecules (termed exerkines), some of which have endocrine functions, with which they can modulate the physiology of even remote organs [27]. Exercise, hypoxia, or hypoxia-related mitochondrial stress may be central triggers for such inter-organ signaling, although experimental evidence for these effects is largely missing [28]. Despite the lack of data in humans, exercise-induced signaling between skeletal muscles (and other tissues) with the brain is a current topic of great interest: the majority of scientific evidence, however, relates to animal models [2],[29]. Recent umbrella reviews of systematic reviews suggest important overall benefits of exercise, for example, on cognition and memory, although these benefits may have been overestimated in specific populations, including healthy people [30],[31], possibly because exercise may more effectively improve brain function in conditions of deficit as compared to highly functional healthy brains. It is noteworthy that exercise can also improve brain structure and plasticity [32],[33], which may counteract the inexorable progression of neurodegenerative processes in disorders like Parkinson's disease, a feature not yet achievable by pharmacological means. In rodents, exercise can also induce adult neurogenesis in neurogenic niches, such as the hippocampus [34], a cerebral exercise outcome that is still debated in humans. Interestingly, hippocampal neurogenesis has also been described as a result of hypoxia in mice [35],[36].

**Figure 1.**
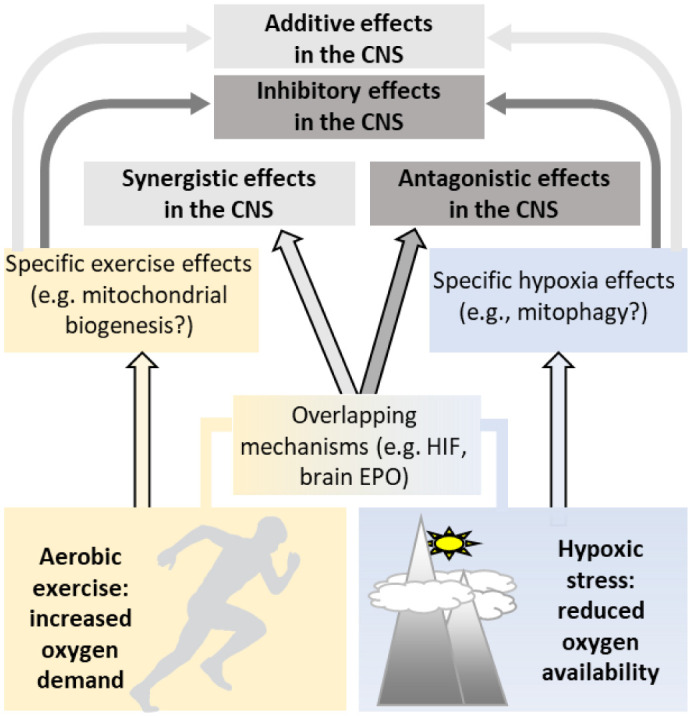
Different outcomes of the combination of exercise and inspiratory hypoxia.

Taken together, it is possible that hypoxia, resulting from increased oxygen demand, may partly mediate the systemic benefits of exercise. While it can be assumed that ambient hypoxia can be used to increase the hypoxic stress of exercise to boost hypoxia-related beneficial adaptations, including in the brain, too much hypoxia can clearly also have adverse effects by overwhelming the innate hypoxia response mechanisms (Figure 1). Both exercise and exposure to ambient hypoxia induce a set of overlapping hypoxia responses [37], including the activation of HIF pathways, which—*inter alia*—may regulate the expression of erythropoietin (EPO), a main mechanism of altitude training, improving oxygen delivery, as discussed in more detail below. But exercise and hypoxia also induce relatively independent effects, such as the promotion of mitochondrial biogenesis (by aerobic exercise) and improved mitochondrial clearance or mitophagy (by hypoxia, although the evidence in humans is still low, especially in the brain) [37]. These processes are complementary in health but may also antagonize each other in conditions of deranged metabolic homeostasis.

## 4. Combination of exercise and high-altitude hypoxia: learnings from competitive sports

High altitude is characterized by reduced atmospheric pressure and, therefore, also a partial pressure of oxygen (hypobaric hypoxia). Although it has long been acknowledged that these conditions impair endurance performance, this problem became particularly interesting before the 1968 Olympic Games in Mexico [38]. With most competitions taking place in or around Mexico City, at an average altitude of 2239 m above sea level, it was expected that this would compromise the aerobic capacity of the athletes. The average partial pressure of oxygen (PO_2_) of 120 mmHg at 2239 m reflects a 25% reduction of sea level PO_2_ of 160 mmHg (at standard dry air). These conditions had pronounced negative effects on race times in long-distance—but not in short—running disciplines. Table 1 shows selected sprint and endurance events of the 1968 Olympic Games in Mexico.

**Table 1.** Official records of selected running events in the 1968 Olympic Games in Mexico and calculations of slower times as compared to previous world records (WR) in long-distance events of men (there were no long-distance running events above 800 m for women at these Olympic Games).

Event	Winning time	WR?	Previous WR	Change (%) compared to the previous WR
100 m	9.95 s (Hines, USA)	Yes	10.03 s	−0.7%
200 m	19.83 s (Smith, USA)	Yes	19.92 s	−0.5%
400 m	43.86 s (Evans, USA)	Yes	44.19 s	−0.8%
5000 m	14:05.01 (Gammoudi, TUN)	No	13:16.6	5.8%
10000 m	29:27.40 (Temu, KEN)	No	27:39.4	6.1%
Marathon	2:20:27 (Wolde, ETH)	No	2:09:36	7.9%

The notoriously slow times in long-distance running events, while many world records were broken at sprint events, can readily be explained by the preferential energy metabolism pathways for exercise with different temporal energy requirements [39]. Skeletal muscle can operate on stored ATP and creatine phosphate for up to about 15 s, without the need for oxygen. Non-oxygen-dependent glycolysis can provide sufficient ATP for longer (up to approximately 2–3 min) high-intensity physical efforts by breaking down glucose [40]. For longer duration exercise (aerobic), muscles rely on oxygen-dependent oxidative phosphorylation, which is more efficient than glycolysis (producing more molecules of ATP per molecule of glucose used), and can also use other substrates aside from glucose (e.g., fatty acids) for ATP production. Therefore, at higher altitudes, where less oxygen is available, aerobic energy metabolism is impaired, but not anaerobic pathways [41]. Nevertheless, no causal relationships can be deduced from these observations, as many potential confounding factors could have contributed to the results of the running competitions at the 1968 Olympic Games in Mexico City. These factors include, notably, athlete selection, environmental conditions other than hypoxia, the track surface, and tactical racing decisions.

Before and after the 1968 Olympic Games in Mexico City, training methods to improve the aerobic capacity of athletes at higher altitudes were investigated, leading to the development of various types of altitude/hypoxic training [13],[42],[43].

It has become clear that pre-acclimatization to hypoxic conditions can substantially mitigate the detrimental effects of subsequent hypoxia. While 1–3 days in hypoxia can progressively reduce the negative effects on performance, whether sufficient time in hypoxia can entirely abolish performance decrements during a later exposure to target altitudes remains under debate [44]. However, hypoxia acclimatization also reduces the risk for high-altitude diseases, like acute mountain sickness (AMS) [45]. Very recently, it has been shown that around 200 hours of well-chosen pre-acclimatization (between 2200 and 5000 m) appear to reduce to virtually zero the subsequent risk for AMS at terrestrial altitude [46]. Since AMS is an illness affecting primarily the brain [47], this indicates that pre-acclimatization is cerebro-protective.

While the evidence for acclimatization effects of high-altitude training clearly supports reduced performance during high-altitude competitions (or non-competitive aerobic exercise), it is less clear how effective altitude training is in increasing performance at low altitude. This is despite a long history of investigation of and the reliance of many athletes and coaches on the effects of high-altitude training [48],[49]. Several potentially beneficial mechanisms are discussed that could improve low altitude performance and may—in parallel—even confer general health benefits that are currently being explored for clinical applications. The involvement of adaptive effects in the brain is likely [10],[50] and could represent an important additional, hitherto largely overlooked, outcome of altitude training.

## 5. Altitude training types and possible mechanisms

Much research is available on altitude training. Here, we discuss what is known about involved mechanisms, potentially adding to our understanding of how and why certain training types can influence brain function and induce potentially beneficial adaptations. A wide variety of altitude training modalities based on diverse methods have been described and are used by athletes [51]. The first protocols involved living and training (live high/train high) usually for 2–3 weeks at moderate altitude [52]. Due to the reduced performance capacity at high altitude, a logistically more demanding strategy was explored, in which living high (mainly sleeping) was combined with training sessions at normal intensities at low altitude (live high/train low) [53],[54]. This strategy was shown to be superior to live high/train high designs in competitive runners [53],[54]. Later variations combined living high with combinations of training at low and moderate altitudes. While live high/train high has been practiced primarily at moderate terrestrial altitudes, variations combining moderate/high with low altitude often use hypoxia-generators or gas mixes (hypoxia chambers or tents, hypoxic gas mixes inhaled through face masks). The main assumed performance-enhancing mechanisms of living high (in combination with both training high or low) relate to hematological adaptations, involving upregulation of EPO, increased hemoglobin levels, and red blood cell mass [55]. Increased hemoglobin levels are thought to result in increased exercise performance (e.g., power output or VO_2_ max). This direct relationship between high-altitude training-induced increases in hemoglobin and VO_2_ max has not been observed consistently, resulting in debates on the use of high-altitude training, especially in elite athletes [48],[49]. Studies on large populations of elite endurance athletes, however, found a clear correlation between increased hemoglobin levels after different high-altitude training types and VO_2_ max [56]. Although the variation of hemoglobin responses in hypoxia is large [48], adequate altitudes are expected to yield approximately 1% increases of hemoglobin per 100 h of exposure [57], not normally exceeding a maximum of 3%–4% in total. It is still being investigated which altitudes are most adequate for high-altitude training, but common recommendations are 1800–2500 m for live high/train high strategies, while considerably higher altitudes (or more severe hypoxia) are frequently chosen for live high/train low or live low/train high protocols [58]. A recent study [59] compared hematological adaptations in two live high/train high training camps (both 3 weeks long) of professional cyclists at either 2050 or 3000 m. Average increases in hemoglobin mass of 3.7% at 2050 m and 3.5% at 3000 m confirm that higher altitudes likely do not yield additional hematological benefits.

Hematological adaptations are not the only factors potentially improving performance at low altitude in live-high strategies. For example, improvements in running economy and metabolic regulation (e.g., lactate thresholds) have also been suggested. Moreover, hormonal changes may underline high-altitude training benefits. This is, for example, indicated by increased serum testosterone and insulin-like growth factor-1 levels following live high/train high in professional cyclists [59]. However, effects on the brain, which have not yet been given much consideration, may play a role as well. It has been suggested that the brain EPO cycle can explain how the brain undergoes an adaptive “hardware upgrade”, resulting in improved performance [60], as discussed in more detail below.

Furthermore, changes in perceived effort and performance following altitude training suggest underlying neurophysiological and psychological adaptations to exposure to hypoxia [50]. Furthermore, an increase in red blood cells related to training in hypoxia may improve systemic glucose metabolism [61]. This could have a beneficial impact on brain function and represent a novel therapeutic approach for hyperglycemic disorders as well [61].

Many contemporary hypoxic training concepts involve relatively short hypoxia exposures, usually combined with exercise but sometimes also at rest, while living at low altitude [51],[58]. In these strategies, athletes are exposed to (simulated) altitudes for only several minutes to a few hours daily or some days per week (for several weeks), often intermittently [58]. While traditionally such approaches are not thought to increase EPO levels substantially, recent evidence suggests that especially the intermittent exposure could facilitate circulating EPO upregulation, even if the duration of hypoxia exposure is shorter than necessary for EPO upregulation in continuous hypoxia [62],[63]. Still, potential benefits such as improved metabolic processes (including energy metabolism efficiency or clearance of waste products) or positive effects on fatigue/effort perception [50] probably play more important roles in these strategies. This suggests that these training forms, with their short hypoxia exposures, also likely affect brain function, such as central regulation of fatigue. Moreover, hypoxic exercise might increase energy metabolism and stimulate waste removal in the brain, which could be relevant for many neurological diseases. Such diseases include dementia, like Alzheimer's disease, or movement disorders, like Parkinson's disease, which are characterized by impaired energy metabolism and the accumulation of aggregated proteins and other waste products in the brain [33]. These possibilities remain to be investigated in humans.

Importantly, not only are the mechanisms underlying potential effects on the brain in live-low (and hypoxia conditioning) strategies not well understood yet, but also how effective they are to improve physical performance—the primary aim of altitude training—remains to be conclusively demonstrated. The general metabolic improvements and benefits regarding fatigue make such hypoxic training methods also interesting for athletes from non-endurance sports [49]. Moreover, benefits beyond athletic capacities of hypoxia exposures, especially in combination with exercise, are increasingly acknowledged [7]. Although research on altitude training and on therapeutic hypoxia modalities (hypoxia conditioning) has resulted in the development of separate research fields with little crosstalk, the overlap of biological and psychological effects is obviously large, and an evaluation of investigated and suggested molecular and physiological mechanistic underpinnings is of interest for both research fields. In particular, the effects of athletic altitude training on the brain are surprisingly poorly investigated, although many studies on both passive hypoxia and hypoxia combined with exercise in clinical settings have been published and recently reviewed [12],[64],[65]. Therapeutic hypoxia conditioning has evolved independently of altitude training methods. While altitude training focuses on enhancing athletic performance, therapeutic hypoxia conditioning approaches focus on health benefits. The potential health benefits of these strategies are discussed in the following section, with a particular focus on the brain.

## 6. Clinical applications of exercise and hypoxia combinations: focus on neuro-psychiatric diseases

Starting from investigations of cardiovascular and neurological benefits of hypoxia exposures in the former Soviet Union [66], therapeutic hypoxia strategies as diverse as altitude training types have developed [7],[67]. Often, they are based on specific mechanistic assumptions that may be particularly relevant for the respective methods.

Unlike for altitude training, only a few studies have investigated long-term (days to weeks) exposure to hypoxia/altitude for clinical applications in humans. Instead, similar to how continuous altitude training (life high/train high) has transitioned toward intermittent variations, a transition fueled by technological advances in the generation and administration of simulated altitude (hypoxic chambers, controlled delivery of gas-mixtures via facemasks, etc.), therapeutic hypoxia in humans has also been used primarily intermittently. These strategies vary significantly in terms of hypoxia intensity, duration, frequency, and patterns of exposure [7]. Moreover, therapeutic hypoxia is frequently applied passively, i.e., not combined with exercise. Both passive and active intermittent hypoxia protocols can improve physical performance if appropriate doses are chosen. A recent umbrella review of systematic reviews and meta-analyses concluded that aerobic and anaerobic performance and skeletal muscle strength can all be improved by intermittent hypoxia protocols, whether they are combined with exercise or not [68]. Aerobic capacity could be increased not only with live high/train low and live low/train high–type protocols, but also with intermittent hypoxic interval training and high-intensity interval training under hypoxia. Repeated sprint training in hypoxia is assumed to reduce sprint-fatigue resistance and improve glycolytic capacity [68], thus probably relying primarily on very different mechanisms than the hematology-oriented altitude training strategies.

**Figure 2.**
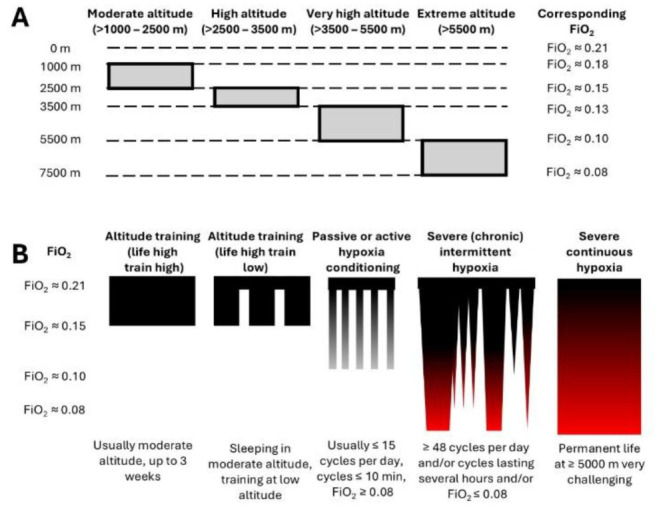
Altitude levels (A) and different types of hypoxia exposure (B). (A) Acute physiological responses to hypoxia depend on altitude.

It is crucial to note that the hypoxia protocols currently investigated are highly diverse, impeding a clear synthesis of published findings, due to a multitude of factors, such as type of protocol (e.g., intermittent versus continuous, active versus passive), hypoxic dose (in active protocols, exercise dose), and individual factors (training, dietary, health, regeneration status, etc.). However, many mild hypoxic intermittent hypoxia protocols (for a comparison of mild therapeutic versus severe pathogenic intermittent hypoxia protocols, see [69]) and moderate continuous hypoxia exposure appear to be safe and may induce similar hypoxia responses and protective adaptations [70]. Yet, even subtly different hypoxia protocols may differ in certain molecular and systemic responses, and also when applied to different populations and individuals. For instance, longer daily exposure to intermittent hypoxia appears to increase the ventilatory response to hypoxia (HVR) to a greater extent than shorter exposure periods [71],[72]. Conversely, short-term intermittent hypoxia (e.g., 5 minutes of hypoxia interspersed with 5-minute periods of normoxia) may result in earlier HVR peak responses and slower HVR decline following the intervention [71],[72]. Also, time of administration, sex, race, age, and carbon dioxide levels are all factors whose influence still needs to be investigated more systematically [73]. Moreover, the type of hypoxia (hypobaric versus normobaric) may determine differential physiological responses, including in the brain [74]–[76]. For example, one study found that, unlike hypoxia, acute hypobaria per se may affect postural stability [77]. A systematic review comparing the effects of hypobaric and normobaric hypoxia exposure also revealed slight differences in the responses of some physiological variables (e.g., minute ventilation and nitric oxide levels) [78]. However, the authors emphasized that confounding factors such as time spent in hypoxia, temperature, humidity, and sample sizes may limit the conclusions that can be drawn [78]. Nevertheless, cross-modality conclusions from different hypoxia interventions must be interpreted cautiously. A comparison of different hypoxia exposure and altitude training protocols, highlighting differences in the hypoxic dose, is presented in Figure 2.

Exposure to altitude is associated with time-dependent and individual acclimatization processes [79]. With increasing terrestrial altitude, the barometric pressure and partial pressure of oxygen decrease, while the relative composition of air changes minimally, causing hypobaric hypoxia. Many experimental hypoxia exposures rely on normobaric hypoxia, characterized by a reduced fraction of inspired oxygen (FiO_2_). The outcomes of hypoxic exposure depend strongly on the exposure protocol. Moderate altitude exposure is well tolerated by humans and is frequently used in altitude training. Passive or active exposure to intermittent hypoxia within the indicated ranges is considered safe and can promote health (hypoxia conditioning) [7],[80]. More severe intermittent hypoxia (e.g., as occurring in obstructive sleep apnea) can damage the brain and induce pathologies [64]. While living at moderate altitudes has been associated with mortality benefits [81], adapting to altitudes above 5000 m is challenging for humans [82].

In the following, we focus primarily on repeated (as opposed to single) intermittent hypoxia interventions, which have emerged as particularly promising for human brain health. Such medium- or long-term intermittent hypoxia interventions (at least 3 times per week for several weeks) have also recently been found to be more promising than acute interventions regarding various health-related outcomes, such as body composition, metabolism, cardiovascular system, exercise performance, or quality of life [83].

It is becoming increasingly clear that passive exposures to intermittent hypoxia can partly mimic general benefits of regular aerobic exercise in humans, such as improved exercise tolerance [84]–[88], aspects of cardiorespiratory fitness such as peak ventilation [88]–[90], autonomic control [91], vascular function [89], as well as reduced blood pressure [89],[92], metabolism [93], body composition [94], and inflammation [95] (see Table 2 for study characterizations). These benefits of passive hypoxia alone suggest that hypoxia, as an isolated component of exercise, may be involved in the health-promoting outcomes of regular exercise and, more specifically, in the brain benefits of exercise [37]. Specifically, aerobic exercise is associated with an increased oxygen demand, especially to satisfy the increasing energetic demand of working muscles and heart, which results in local (tissue) hypoxia. The physiological responses, described in more detail above, involve increased ventilation and transport of blood to these tissues but also to the brain. Certain passive hypoxia protocols have been reported to be similarly effective in promoting, for example, cardiorespiratory fitness like exercise interventions in specific populations (Table 2).

**Table 2.** Selected studies reporting exercise performance-related benefits of passive intermittent hypoxia.

Subjects	Intervention	Results	Refs
22 older participants were assigned to hypoxia (71.2 ± 6.1 years) or a sham control group (71.7 ± 5.3 years)	6 cycles of 5 min of hypoxia (SaO_2_ = 85%–92%) followed by 3 min of hyperoxia (SaO_2_ = 95%) per session, 3 times per week for 6 weeks	Significantly improved respiratory (increased maximal inspiratory pressure variable), heart rate variability (decreased low frequency to high frequency ratio), and inflammatory biomarkers (decreased C-reactive protein) outcomes only in the hypoxia group	[95]
26 older participants (60–80 years) were assigned to hypoxia (N = 12) or a sham control group (N = 14)	24 passive hypoxia sessions (3/week), each session consisting of 7 × 5 min cycles (target SaO_2_ = 80%–75%), each followed by 3 min normoxia	During cardiopulmonary exercise testing, peak ventilation improved significantly in the hypoxia group, but no other parameters did (e.g., VO_2_ peak, work rate, heart rate, respiratory rate, leg discomfort, dyspnea, etc.)	[89]
145 patients with long COVID (74% female, 53 ± 12 years) were assigned to IHHT (n = 70) or standard care (n = 75)	The IHHT group received intermittent hypoxia (F_I_O_2_ = 0.10–0.12) and hyperoxia (F_I_O_2_ = 0.30–0.35) 3× per week throughout rehabilitation	Walking distance in a 6-min walk test improved 2.8-fold and stair climbing power 3.7-fold in the IHHT versus control group. Dyspnea, fatigue, and quality of life improved significantly in IHHT compared to controls. Blood pressure and heart rate decreased significantly, and hemoglobin levels increased in IHHT versus control	[86]
70 obese participants (BMI >30 kg/m^2^, 40–75 years) assigned to IHHT (N = 35) or sham (N = 35), all received balneo-physical-kinetic treatment	The IHHT group received 9 sessions of intermittent hypoxia (F_I_O_2_ = 0.09–0.16) and hyperoxia (F_I_O_2_ ≈ 0.35) for 2 weeks	Significant increases in exercise tolerance, renal function (uric acid, creatinine), and liver function (alanine aminotransferase and aspartate aminotransferase) markers in IHHT versus control	[87]
Cardiology outpatients: hypoxia group (N = 15, 66.7 ± 5.7 years) or sham control group (N = 14, 65.0 ± 6.2 years) that received additional exercise interventions	The passive IHHT consisted of 5–7 × 4–6 min F_I_O_2_ = 0.12–11, interspersed with 3 min F_I_O_2_ = 0.3–0.33 for 5 weeks; the control group followed an 8-week exercise intervention	Both the passive hypoxia intervention for 5 weeks and the exercise intervention for 8 weeks similarly improved cardiorespiratory fitness	[90]
27 coronary artery disease patients (52–77 years) received IHHT, 19 (43–83 years) patients received sham	IHHT consisted of 15 sessions (3/week) of 5–7 × 4–6 min F_I_O_2_ = 0.12–0.10, interspersed with 3 min F_I_O_2_ = 0.30–0.35	Patients in the hypoxia group improved exercise capacity, reduced blood pressure and glycemia, enhanced left ventricle ejection fraction, and better rated quality of life	[88]
18 patients (33–72 years) at risk for or with mild COPD, 50% assigned to hypoxia, 50% to normoxic sham control	15 sessions (within 3 weeks) of intermittent hypoxia with 3–5 hypoxic periods of 3–5 min (F_I_O_2_ = 0.15–0.12), interspersed with 3 min normoxia	The hypoxia intervention significantly increased total hemoglobin mass (4%), total exercise time (9.7%), and the exercise time to the anaerobic threshold (13%) compared to controls	[85]
8 healthy people and 8 with myocardial infarction (50–70 years), 50% of each group assigned to hypoxia, 50% to normoxic sham control	The hypoxia group received 5 × 3–5 min F_I_O_2_ = 0.14–0.10, 3-min normoxic intervals in between; 15 sessions in 3 weeks	Similar benefits for healthy people and those with myocardial infarction: increased peak oxygen consumption compared to normoxic conditions, tightly correlated to enhanced arterial oxygen content after hypoxia, reduced heart rate, systolic blood pressure, blood lactate concentration, rating of perceived exertion during sub-maximal exercise	[84]

Notes: F_I_O_2_: fractional inspired O_2_; IHHT: intermittent hypoxia hyperoxia training; SaO_2_: blood oxygen saturation.

Moreover, the combination of inspiratory hypoxia with exercise interventions can enhance general exercise benefits, as shown in athletes, generally healthy people, and various patient groups (see Table 3). Diverse effects can occur in the presence or absence of hematological effects of the interventions. Aerobic exercise interventions have been more frequently investigated in combination with hypoxia, especially intermittent hypoxia [96],[97]. However, there are a few indications that hypoxic conditions during resistance training may modulate strength and muscle growth as well. For example, performing an 8-week resistance training in normobaric hypoxia (F_I_O_2_ = 0.159) led to statistically significant greater strength improvement than in normoxia [98]. On the other hand, muscle thickness increased during normoxia and hypobaric hypoxia (terrestrial altitude of 2320 m, comparable to the normobaric hypoxic conditions), but not in normobaric hypoxia. More research on the effects of the combination of resistance training with hypoxia is required, particularly since resistance training has emerged as a potent factor for promoting brain health and a possible preventive factor for age-related neurological diseases [32],[99],[100].

**Table 3.** Selected studies reporting increased benefits of exercise if combined with intermittent hypoxia.

Subjects	Intervention	Results	Refs
35 participants with chronic stroke were randomized to AIH or normoxic control	Up to 15 sessions of AIH for 30 min using 15 cycles of hypoxia (60–90 s F_I_O_2_ = 0.08–0.09) and normoxia (30–60 s; F_I_O_2_ = 0.21), followed by 1 h of high-intensity gait training (>75% heart rate reserve) for 5 weeks	AIH resulted in greater self-selected speeds, fastest speed, and peak treadmill speed compared to the normoxic control	[101]
Healthy sedentary men and women: normoxic group (N = 18, 56.4 ± 6.5 years), hypoxic group (N = 16, 56.7 ± 6.4 years)	Participants performed the same strength and aerobic exercise training (1 h on 3 days/week, for 10 weeks) together, the hypoxia group also received 6 × 5 min 90%–80% SpO_2_, interspersed by 5 min normoxia, for 10 weeks	Hypoxia improved exercise benefits on blood pressure, cardiorespiratory fitness, and lipid profile (high-density lipoprotein), but might have had negative effects on arterial wave reflection and total cholesterol	[97]
15 athletes with overtraining syndrome, 19 healthy athletes in the control group (18–20 years)	6–8 cycles of 5–7 min F_I_O_2_ = 0.11, interspersed with intervals of F_I_O_2_ = 0.30, total time 45 min–1 h), 3× week, delivered 1.5–2 h after a low-intensity exercise session (2 bouts of 30 min, running at 50% of VO_2_ max with 10 min rest between bouts) over 4 weeks	Improved exercise performance and sympatho–parasympathetic index in the athletes with overtraining syndrome, without changes in hematological parameters	[96]

Notes: AIH: acute intermittent hypoxia; F_I_O_2_: fractional inspired O_2_; IHHT: intermittent hypoxia hyperoxia training; PETCO_2_: partial pressure of end‑tidal CO_2_; PETO_2_: partial pressure of end-tidal O_2_; SaO_2_: blood oxygen saturation

As may be expected from the potential mechanistic overlaps of health benefits from inspiratory hypoxia and exercise, as demonstrated in preclinical studies and the well-evidenced potential of passive hypoxia to mimic some of the general exercise benefits in humans, hypoxia can be used to promote brain health. This includes cognition and psychological parameters, especially in older individuals [12]. Exemplary studies supporting such effects are presented in Table 4.

**Table 4.** Studies on intermittent hypoxia conditioning and outcomes related to human brain functions and mental health.

Subjects	Hypoxia intervention	Exercise	Main results	Refs
50 healthy participants in the hypoxia group (36 ± 7 years), 50 healthy participants in the sham group (35 ± 6 years)	2 sessions/day of 4 × 10 min cycles of F_I_O_2_ = 0.13, interspersed with 5 min normoxic cycles, for 5 days	No	No differences in cognitive tests; improved cerebral blood flow after hypoxia	[102]
16 patients with MCI and 13 age-matched healthy elderly subjects (52–76 years) were assigned to one of the following groups: healthy + sham (N = 7), healthy + hypoxia (N = 6), MCI + sham (N = 6), or MCI + hypoxia (N = 10)	4 cycles of 5 min hypoxia (F_I_O_2_ = 0.12) interspersed with 3 min periods of hyperoxia (F_I_O_2_ = 0.30), 5 days/week for 3 weeks (15 sessions in total)	No	Slightly improved MoCA scores in the MCI + hypoxia group on the first day after hypoxia. Improved latency of cognitive evoked potentials in the hypoxia group only	[103]
48 subjects with migraine (5 men, 43 women, 31.3 ± 7.8 years)	5 × 5 min cycles/session (F_I_O_2_ = 0.10), interspersed with 5 min room air; 5×/week, for 8 weeks and control group	No	Reduced migraine frequencies within 3 months after hypoxia but not normoxic control; depressive (BDI) and anxiety (BAI) symptoms improved only after hypoxia	[104]
7 patients with amnestic MCI, all exposed to hypoxia (69 ± 3 years)	5 min F_I_O_2_ = 0.10, interspersed with 5 min normoxia; 8 cycles/session, 3 sessions/week for 8 weeks	No	Improvements in mini-mental status exam and digit span scores, increased cerebral vasodilation, and tissue oxygenation after hypoxia	[105]
25 geriatric patients were separated in a hypoxia (N = 14, 84 ± 5 years) or normoxia sham group (N = 11, 86 ± 6 years)	Intermittent hypoxic (F_I_O_2_ = 0.10–0.14, 1–5 min) and hyperoxic (F_I_O_2_ = 0.30–0.40, 1–3 min) periods for 30 min sessions prior to exercise (3×/week for 6 weeks)	6 weeks of aerobic training 3×/week for 20 min per day (both groups)	No significant interaction effect for Dementia Detection Test, medium effect size for improved performance of the IHHC group in the clock drawing test	[106]
34 multimorbid geriatric patients were assigned to a hypoxia group (N = 18, 81 ± 8 years); 16 to a normoxia sham group (83 ± 6 years)	4–6 min F_I_O_2_ = 0.12, interspersed with 1–2 min F_I_O_2_ 35% (IHHC), 2–3×/week, 15–16 sessions in 5–6 weeks	Multimodal rehabilitation (2–3×/week, 16–20 sessions in total, both groups)	Improved cognitive performance (dementia test and clock drawing test) only in the hypoxia group	[107]
33 spinal cord injury patients were assigned to the hypoxia group (N = 17, 41 ± 17 years) or to the normoxia sham group (N = 16, 42 ± 17 years)	15 cycles/day with 90 s intervals at F_I_O_2_ = 0.12, interspersed with normoxic periods for 5 consecutive days, then 3× per week for 3 weeks	Body weight-supported treadmill training for 4 weeks (both groups)	Improved verbal memory performance only in the hypoxia group. No difference in the Rey–Osterrieth Complex Figure Test (visual memory, visuospatial constructional ability)	[108]
Healthy, inactive participants; 17 in the hypoxic group (64 ± 3 years), 17 in the normoxic group (64 ± 3 years)	10 min NH interspersed with 5 min normoxia: 4 cycles per day, aiming at reducing SpO_2_ stepwise: 90% in weeks 1–2, 85% in week 3, and 80% in weeks 4–6)	Both groups performed regular 30-min full body strength-endurance training for 6 weeks	Improved cognitive performance of the hypoxic group, indicated by significant differences in the d2 test (processing speed and attention), no significant difference in the Number Combination Test, potential “anti-depressant-like” profile	[109]

Notes: BAI: Beck Anxiety Inventory, BDI: Beck depression inventory; BSI: brief symptom inventory; ERP: event-related potential; FIO2: fractional inspired O_2_; HAMD: Hamilton Depression Rating Scale; MCI: mild cognitive impairment (mild cognitive disorder in DSM-V); MoCA: Montreal Cognitive Assessment test (MoCA); RCT: randomized controlled trial; SpO2: O_2_ saturation.

Acute mild hypoxia exposures can have positive effects on brain functions and mood, an outcome that probably relates to the well-investigated acute physiological responses to both exercise and hypoxia: increased cerebral blood flow, increased ventilation, and increased heart rate. Regarding mood, this has recently been shown in a study conducted in healthy participants exposed to 10 min of (continuous) F_I_O_2_ = 0.135, which resulted in improved cardiac vagal indices and improved mood [110]. In another study [111], a single cycle of 12 intervals of 5-min hypoxia (end-tidal PO_2_ = 50 mmHg), interspersed with 5-min periods of normoxia (end-tidal PO_2_ = 100 mmHg), was shown to improve cerebral blood flow and executive function (tested with the antisaccade task, in which subjects must voluntarily move their gaze toward a specified target upon a visual cue). Single intermittent hypoxia sessions, however, appear to be less effective for improving cognitive function, especially in young and healthy participants. A recent study on healthy subjects aged between 18 and 40 years did not find a superiority of one intermittent hypoxia intervention (8 × 5 min F_I_O_2_ = 0.12, interspersed with 2 min normoxia) as compared to a normoxic control concerning cognitive outcomes [112]. Another study did not find effects of a single session of 5 cycles of 3 min F_I_O_2_ = 0.10 and 2 min normoxia on motor cortical excitability [113].

One special type of therapeutic hypoxia has been termed therapeutic acute intermittent hypoxia (tAIH), which originally consisted of only one session of short (30–120 s) intervals of hypoxia (often of F_I_O_2_ = 0.08–0.12), interspersed with normoxia. More recently, however, such protocols have been increasingly administered repeatedly, similar to other therapeutic intermittent hypoxia protocols. A prominent example are studies on people with chronic stroke, including a recent phase II double-blind randomized, crossover trial, that demonstrated superiority of high intensity gait training outcomes in combination with tAIH as compared to normoxic controls [101] (see Table 3). Moreover, tAIH protocols are being employed in ongoing clinical trials in the context of other neurological diseases (e.g., amyotrophic lateral sclerosis [114]). The main reported benefits in preclinical models are the induction of neuroplasticity leading to ventilatory and motor facilitation, with primary benefits for spinal cord injury, stroke, and neuromuscular diseases [115]. First results from a preprint confirm the preclinical data and suggest benefits, for example, in multiple sclerosis patients, including improved cognitive processing speed [116].

A recently concluded study [117] of randomized controlled multiple N-of-1 trials subjected 20 patients with Parkinson's disease to five different hypoxia interventions (in duplicate, 45 min each): continuous hypoxia (F_I_O_2_ = 0.163 and F_I_O_2_ = 0.127), intermittent (5-min intervals of F_I_O_2_ = 0.163 and F_I_O_2_ = 0.127, interspersed with normoxia), and placebo. While safety and feasibility for longer interventions were confirmed, only the intermittent hypoxia intervention F_I_O_2_ = 0.163 mildly improved participant-rated symptoms compared to placebo. Assessor-rated scales were not improved in any intervention. A repeated intermittent hypoxia strategy based on these findings (5-min F_I_O_2_ = 0.163, interspersed with 5 min normoxia, 45 min per session, 3× per week for 4 weeks) is ongoing [118]. The results may show if hypoxia conditioning is really as promising a treatment strategy in Parkinson's disease as has been proposed [119]–[121].

Commonly used hypoxia protocols reporting positive brain or mental health effects consist of repeated exposures to short intervals (about 1–10 min long) of hypoxia (F_I_O_2_ often 0.10–0.14), interspersed with equally long or slightly shorter periods of normoxia or mild hyperoxia (frequently F_I_O_2_ between 0.30 and 0.40). A summary of the details of some selected studies of this type is provided in Table 4. Often, the duration and severity of the hypoxic exposure are determined based on the individual hypoxic ventilatory response and tailored to not fall below a certain threshold of blood oxygen saturation (e.g., 80%). Such protocols are thought to protect from brain-related high-altitude diseases like acute mountain sickness and prevent high-altitude-related cognitive impairments, as demonstrated in a recent study [122]. In this study, 24 young male participants underwent one intermittent hypoxia session (8 × 5min to a blood oxygen saturation of 85%, interspersed by 3 min normoxia) on each of 10 days, after which they were exposed to a simulated altitude of 4300 m.

A few small pilot studies report improved cognitive function following isolated (i.e., not combined with exercise interventions) repeated intermittent hypoxia in people with mild cognitive disorder [103],[105]; however, these results were not confirmed in healthy adults in a well-powered RCT, although cerebral blood flow was improved [102]. A similar intervention in people with migraine not only improved migraine but also symptoms of anxiety and depression [104]. Well-designed and well-powered RCTs investigating the potential of passive hypoxia conditioning to improve cognitive function and mood are urgently needed, especially in patient samples suffering from impaired cognition and in patients with psychiatric diseases. Although there is a strong rationale derived from preclinical studies that intermittent hypoxia by itself may be beneficial, for example, in dementia [64] and different psychiatric conditions [123], the evidence in humans is weak.

There is slightly stronger evidence for cognitive benefits when intermittent hypoxia is combined with exercise interventions. In 2013, Schega and colleagues reported cognitive improvements from a pilot study after 6 weeks of regular full-body strength-endurance training, complemented with passive intermittent hypoxia [109]. The same group obtained similar results in a subsequent RCT, in which regular aerobic bicycle training and passive (short, but continuous) hypoxia interventions were performed regularly for 4 weeks [124]. Improvements in verbal memory were seen in spinal cord injury patients, who performed body weight–supported treadmill training and passive intermittent hypoxia for 4 weeks [108]. Two RCTs in geriatric patients convincingly suggest cognitive benefits when passive intermittent hypoxia was combined with rehabilitation training [107] or aerobic exercise [106].

Another strategy currently explored in a randomized clinical trial [125] uses daily motor-cognitive training (participants can choose in which physical or cognitive training they engage, but they have to stay active) in 3.5 h of continuous hypoxia per day for 3 weeks. The premise of this approach is that the combination of inspiratory hypoxia with increased cerebral oxygen demand due to the motor-cognitive activity (“functional hypoxia”) provides a sufficient stimulus for an upregulation of erythropoietin and its receptor in the brain, which may be neuroprotective and improve brain function, but without prominently increased hemoglobin, hematocrit, or red cell mass [126]. Based on a robust preclinical foundation [35],[36], this approach has recently yielded promising results for improving cognition and mood-related parameters such as depressive symptoms [127]. Yet, whether the cerebral EPO effects, as shown in animal models, are also necessary for the cognitive and mental health benefits in humans remains to be demonstrated.

Repeated intermittent hypoxia protocols (often called intermittent hypoxia training/therapy/conditioning or exposure) are increasingly performed with hyperoxic intervals. Preclinical studies in animals suggest that such combined hypoxia–hyperoxia protocols may more efficiently induce beneficial adaptations, in particular strengthening antioxidation [128]. Evidence for this assumed superiority of intermittent hypoxia–hyperoxia versus intermittent hypoxia protocols in humans is largely lacking. However, Behrendt and colleagues [129] recently compared the effects of intermittent hypoxia (F_I_O_2_ = 0.14)–hyperoxia (F_I_O_2_ = 0.30), hypoxia–normoxia, and normoxia alone during 40 min of cycling exercise (submaximal constant-load cycling at 60% peak oxygen uptake). They observed that an exacerbated cardiovascular and metabolic stress of intermittent hypoxia, as compared to normoxia, could be prevented by the addition of hyperoxic intervals. Hyperoxic intervals did not affect the earlier perceived motor fatigue in intermittent hypoxia as compared to normoxia, but improved muscle oxygenation. Importantly, whether the hypoxic conditions objectively improved performance has not been evaluated in this study. Overall, the superiority of adding hyperoxic periods to intermittent hypoxia conditioning protocols compared to traditional protocols remains to be convincingly demonstrated, particularly with regard to effects on the human brain.

In summary, contemporary hypoxia interventions targeting neuropsychiatric diseases do not usually aim primarily at hematological responses, as live-high types of high altitude training do. In particular, cycles of intermittent hypoxia with only several minutes of hypoxia exposure in total probably do not induce prominent hematological changes, although these cannot be excluded in such protocols, as indicated by recent research [62],[63]. Still, depending on the type of the hypoxia intervention, factors like improved molecular resilience against hypoxia, oxidative, inflammatory, and energetic stress, an upregulation of neuroprotective pathways, such as the brain erythropoietin system, the induction of neuroplasticity via oxygen sensing systems, and associated respiratory and motor facilitation, as well as favorable metabolic and cardiovascular responses may more importantly underly brain and mental health benefits of therapeutic hypoxia [7],[115],[126]. Importantly, the roles of molecular pathways and cellular hypoxia responses in human brain benefits of therapeutic hypoxia are poorly understood and need more research.

## 7. Conclusions and recommendations for prioritized research

Altitude training has become an important training modality for athletes for both high-altitude competitions and to improve performance at low altitude. Although the mechanisms and whether all athletes benefit from related training types remain under debate, hematological adaptations to the hypoxic conditions seem to be the primary benefits of live-high strategies for endurance performance enhancement. In other strategies, in which athletes are exposed only briefly to hypoxia (such as in many train-high-type methods), the total hypoxia exposure may not be sufficient to induce hematological changes. The underlying mechanisms are still being evaluated, but the assumed positive effects (for example, on metabolism and fatigue perception) make these training forms interesting for athletes beyond endurance sports.

Effects of athletic altitude training on brain functions have been rarely investigated, but the influence of hypoxia and the associated adaptation of the brain may contribute to improved performance, as suggested by systemic physiological responses to hypoxia and by molecular mechanistic evidence from cell and animal studies, which, for the greatest part, remain to be validated in humans. Among the well-established hypoxia outcomes that contribute to maintaining brain functions and cerebral oxygenation in hypoxia—and may also be beneficial in clinical applications—are ventilatory, cardiovascular, and hematological responses and adaptations. How these outcomes may functionally improve brain function via, for example, reducing fatigue, improving neuronal circuits related to motor performance, or enhancing motivation, is not well understood yet. Molecular mechanisms that may promote brain health remain speculative for humans. Although animal models and findings from human muscle biopsies and blood samples suggest HIFs, redox, and inflammatory regulation, mitochondrial adaptation, neurotrophic factors, and other pathways to be involved, validation for the human brain is largely lacking.

The unclear mechanisms contrast with an increasing number of clinical trials, demonstrating cognitive, mental health, and potential neuroprotective benefits of intermittent hypoxia conditioning approaches, especially if combined with exercise.

Overlapping brain and mental health benefits of both regular exercise and passive exposure to controlled mild hypoxia exist, suggesting exercise in hypoxia and variations of altitude training types to represent potential treatment strategies for neuropsychiatric diseases. In addition, passive intermittent hypoxia for therapeutic purposes may mimic specific aspects of health promotion, including the brain, which could be used as an alternative strategy to regular exercise, for example, in individuals unable or unwilling to engage in sufficient exercise.

Several clinical trials on intermittent hypoxia applications aiming to improve brain functions or neuropsychiatric diseases have recently been concluded or are currently ongoing. This will hopefully increase our knowledge of the potential of hypoxia interventions to improve brain health.

However, many unknowns have yet to be addressed scientifically to better understand the risks and potential of different types of altitude/hypoxia training as clinical applications for brain health. A better understanding is needed to reduce the stigma surrounding the negative effects of hypoxia on the brain and to standardize the terminology and approaches to therapeutic hypoxia. This is particularly important for developing personalized hypoxic treatments tailored to individual needs and vulnerabilities. For the field to advance, answers are needed to inform both mechanistic studies and clinical trials. At the mechanistic level, there is an urgent need for the translational validity of animal model results for humans. To this end, advanced imaging techniques such as functional magnetic resonance imaging and positron emission tomography, as well as approaches to monitor oxygenation (e.g., via near-infrared spectroscopy) or determine molecular processes in the brain (e.g., via cerebrospinal fluid analyses or trans-cerebral exchange of circulating substances, as recently described for inflammatory molecules) can be useful [130].

At the clinical trial level, terminological harmonization is necessary to facilitate the comparison and integration of data. Furthermore, a systematic investigation of different hypoxia strategies (varying in hypoxic dose) is required in order to identify the most effective strategies for specific populations and individuals. Such individualized designs are increasingly being used in recent studies, e.g., on hypoxia conditioning in Parkinson's disease [117]. Another unmet need is the investigation of the duration of beneficial brain effects of hypoxia by sufficiently long follow-up testing designs. It is well-established that the patterns of hypoxia responses change with exposure duration [79] and that acclimatization effects are transient [46]. However, the duration of cognitive or mental health enhancements after hypoxia conditioning and how these effects can be prolonged are unknown. Altitude training may be a particularly useful method of monitoring brain performance in hypoxia. Such data would be valuable in improving our understanding of the effects of hypoxic training on the brain and could lead to recommendations for optimizing and personalizing therapeutic hypoxia applications for brain health.

## Use of AI tools declaration

The authors declare they have not used Artificial Intelligence (AI) tools in the creation of this article.
